# Sugarcane leaf dataset: A dataset for disease detection and classification for machine learning applications

**DOI:** 10.1016/j.dib.2024.110268

**Published:** 2024-02-29

**Authors:** Sandip Thite, Yogesh Suryawanshi, Kailas Patil, Prawit Chumchu

**Affiliations:** a Vishwakarma University, Pune, India; b Kasetsart University, Sriracha, Thailand

**Keywords:** Classification, Dataset, Deep learning, Disease detection, Image analysis, Leaf diseases, Machine learning, Sugarcane

## Abstract

Sugarcane, a vital crop for the global sugar industry, is susceptible to various diseases that significantly impact its yield and quality. Accurate and timely disease detection is crucial for effective management and prevention strategies. We persent the “Sugarcane Leaf Dataset" consisting of 6748 high-resolution leaf images classified into nine disease categories, a healthy leaves category, and a dried leaves category. The dataset covers diseases such as smut, yellow leaf disease, pokkah boeng, mosale, grassy shoot, brown spot, brown rust, banded cholorsis, and sett rot. The dataset's potential for reuse is significant. The provided dataset serves as a valuable resource for researchers and practitioners interested in developing machine learning algorithms for disease detection and classification in sugarcane leaves. By leveraging this dataset, various machine learning techniques can be applied, including deep learning, feature extraction, and pattern recognition, to enhance the accuracy and efficiency of automated sugarcane disease identification systems. The open availability of this dataset encourages collaboration within the scientific community, expediting research on disease control strategies and improving sugarcane production. By leveraging the “Sugarcane Leaf Dataset,” we can advance disease detection, monitoring, and management in sugarcane cultivation, leading to enhanced agricultural practices and higher crop yields.

Specifications Table

**Table:** 

Subject	Applied Machine Learning, Agriculture
Specific subject area	Agronomy & Crop Science
Type of data	Images
How data were acquired	The acquisition of the Sugarcane Leaf Dataset involved capturing images using a high-resolution camera on a mobile phone.
Data format	Raw
Parameters for data collection	The Sugarcane Leaf Dataset comprises images stored in the .jpg format, featuring dimensions of 768 × 1024 pixels. The images possess a resolution of 72 dots per inch (dpi).
Description of data collection	The data collection process encompassed several stages to ensure a comprehensive representation of sugarcane leaf samples. Extensive field surveys were conducted to gather a diverse range of leaves affected by various diseases. High-resolution images of sugarcane leaves were captured using quality cameras, employing multiple angles to capture different perspectives of the leaves. This included capturing images from both sides of the leaves to capture a holistic view of their condition. Furthermore, to facilitate detailed analysis, images were captured in different scenarios. Some images were taken directly in the field, where leaves were in their natural environment, while others were taken after cutting or separating the leaves from the plant. This approach allowed for a more focused examination of the leaf characteristics and disease symptoms, providing a comprehensive dataset that reflects real-world scenarios.
Data source location	**Kendur, Taluka- Shirur, District -Pune** Pin - 412403. Maharashtra, Country- India. Latitude- 18.785097, Longitude- 74.022090
Data accessibility	Repository name: Sugarcane Leaf Dataset Data identification number: 10.17632/355y629ynj.1 Direct URL to data: https://data.mendeley.com/drafts/355y629ynj

## 1 Value of the Data

• *Comprehensive and Diverse:* The dataset comprises 6748 high-resolution images, serving as a valuable resource for studying sugarcane leaf diseases and healthy leaves. It enables effective disease detection and classification in sugarcane.
• *First Open-Access Dataset*: This dataset is the first openly accessible collection of sugarcane leaf samples. It facilitates collaboration among researchers, accelerating advancements in disease detection, monitoring, and management in sugarcane cultivation.
• *Disease Management:* With 11 categories including nine diseases, a healthy leaves category, and a dried leaves category, the dataset covers a wide range of sugarcane leaf conditions. It aids researchers in studying and understanding sugarcane leaf diseases, improving disease detection accuracy, and controlling disease outbreak.
• *Machine Learning Applications:* The dataset's applicability to machine learning algorithms enables automated disease identification systems. With 6748 images, researchers can develop and evaluate models using deep learning, feature extraction, and pattern recognition, enhancing disease detection accuracy in sugarcane leaves.

## 2 Data Description

The image datasets play a crucial role in various fields, ranging from computer vision and machine learning to medical research and social sciences [1]. These datasets provide a rich source of visual information that enables researchers, developers, and professionals to train and validate their models, algorithms, and theories. By having access to diverse and well-curated image datasets, researchers can explore new possibilities, enhance the accuracy and robustness of their models, and gain valuable insights into patterns, trends, and relationships within the visual data [6]. An image dataset specific to sugarcane leaf diseases holds significant importance in the agricultural domain. Such datasets provide researchers, agronomists, and farmers with a valuable resource to identify, classify, and study various leaf diseases affecting sugarcane crops [2]. By analysing these images, experts can develop more accurate disease detection algorithms and early warning systems. This aids in prompt disease management, preventing widespread crop damage and yield loss. Additionally, a comprehensive dataset allows for the exploration of disease patterns, environmental factors, and potential mitigation strategies. In summary, a sugarcane leaf disease image dataset plays a pivotal role in advancing research, improving crop management practices, and ensuring the overall health and productivity of sugarcane crops [3].

**Fig. 1:**
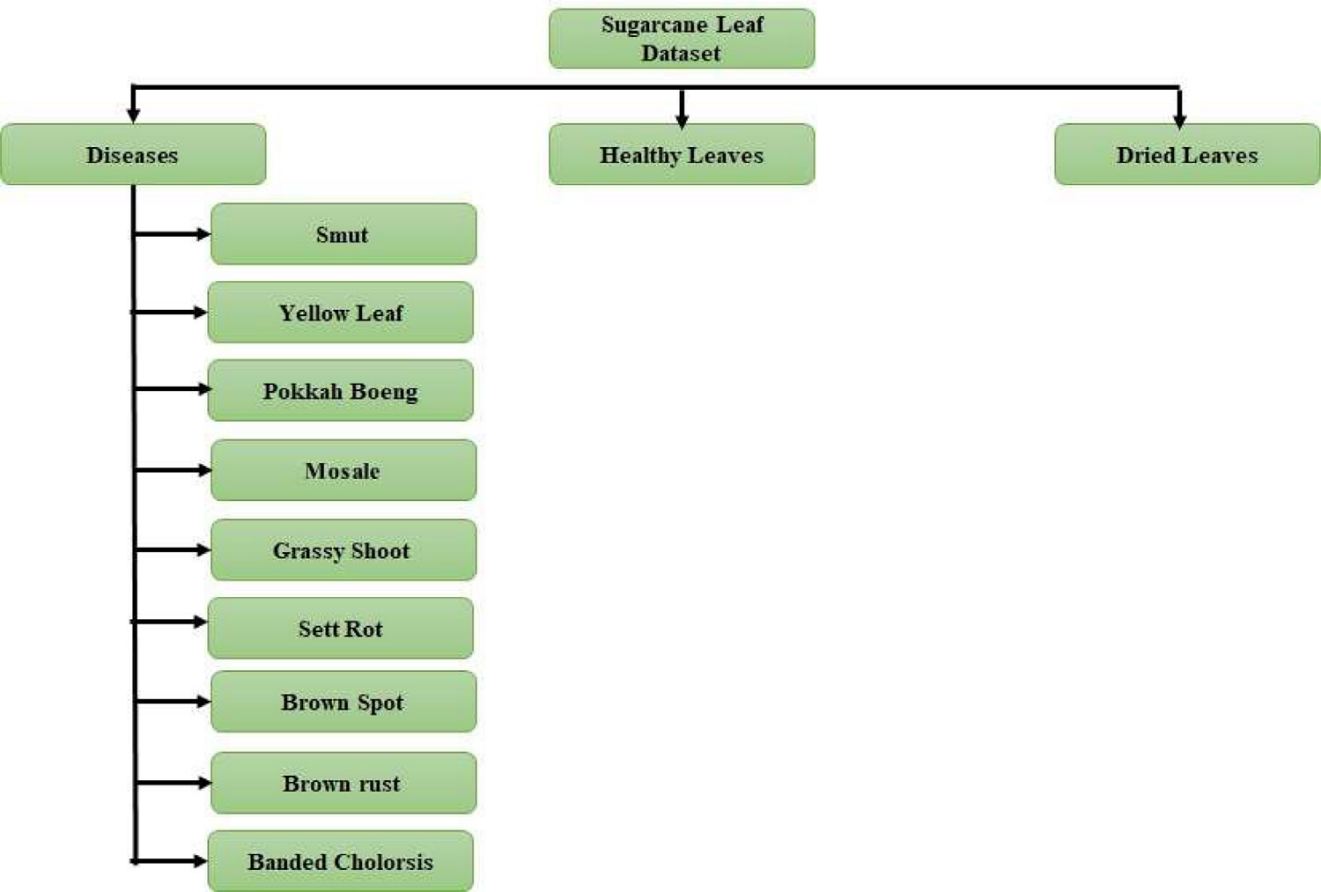
Directory Structure of the Sugarcane Leaf Dataset.

This Sugarcane Leaf Dataset contains a diverse collection of 6748 high-resolution images of sugarcane leaves. The images are stored in JPEG format and have dimensions of 768 × 1024 pixels. The dataset is categorized into 11 distinct classes, including nine disease categories, a healthy leaves category, and a dried leaves category (Fig. 1). The disease categories cover a range of common sugarcane leaf diseases, such as smut, yellow leaf disease, pokkah boeng, mosale, grassy shoot, brown spot, brown rust, banded cholorsis, and sett rot (Table 1). Each category is labelled and organized in separate folders, ensuring easy access and identification of specific disease samples. The images were collected through extensive field surveys conducted in sugarcane-growing regions. The data collection process involved using quality cameras to capture images from various angles, including both sides of the leaves. Images were taken in the field and by cutting/separating individual leaves, capturing different stages and manifestations of the diseases. This approach ensures a comprehensive representation of the visual characteristics of sugarcane leaf diseases within the dataset. The dataset's images are of high quality, with a resolution set at 72 dots per inch (dpi), ensuring clear and detailed visual representation of the sugarcane leaf samples.

**Table 1:** Sample images of different sugarcane leaf (Diseases, Healthy, Dried).

	
**Smut**	**Yellow leaf disease**
	
**Pokkah boeng**	**Mosale (Viral)**
	
**Grassy shoot**	**Sett Rot**
	
**Brown Spot**	**Brown Rust**
	
**Banded Cholorsis**	**Dried Leaf**
	
**Healthy Leaf**	

## 3 Experimental Design, Materials, and Methods

### 3.1 Experimental design

The Sugarcane dataset was generated through the acquistion of images using high resolution rear cameras of Samsung F23 5 G Mobile. The Table 2 provides a summary of the data acquisition steps undertaken for the project.

Step 1: Image Acquisition (Duration: April to June): During this period, field/farm visits were conducted during daytime to capture images. The objective was to gather a collection of images related to sugarcane leaf diseases.
Step 2: Image Pre-processing (Duration: June): In this step, the gathered images were reviewed, and the appropriate images for the dataset were selected. These selected images then underwent pre-processing, which may have included resizing, cropping, and enhancing the images as necessary.

**Table 2:** Data acquisition steps.

Sr. No.	Step	Duration	Activity
1.	Image Acquisition	April to June	During daytime field/farm visits to capture images.
2.	Image Pre-processing	June	The images appropriate for dataset were selected from gathered images and were pre-processed.

The data acquisition process involved capturing images during field visits and subsequently preparing the images for inclusion in the dataset through pre-processing.

### 3.2 Materials or specification of image acquisition system

The cameras used in the data acquisition process and the specifications of the captured images:

1. For Samsung Galaxy F 23 5 G Android Mobile:
  • Make and Model: Samsung Galaxy F 23 5 G (SM-E236B) Android Mobile.
  • Rear Primary Camera: It has a 50-megapixel (f/1.8) lens.
  • Camera Sensor: The camera sensor used is Sony IMX 582 1/2″.
  • Battery: The mobile is equipped with a 5000 mAh battery.

The captured images were saved in JPG format and resized with a resolution of 768 × 1024 pixels. These specifications provide essential information about the cameras and image properties utilized in the data acquisition process.

## 4 Method

**Fig. 2:**
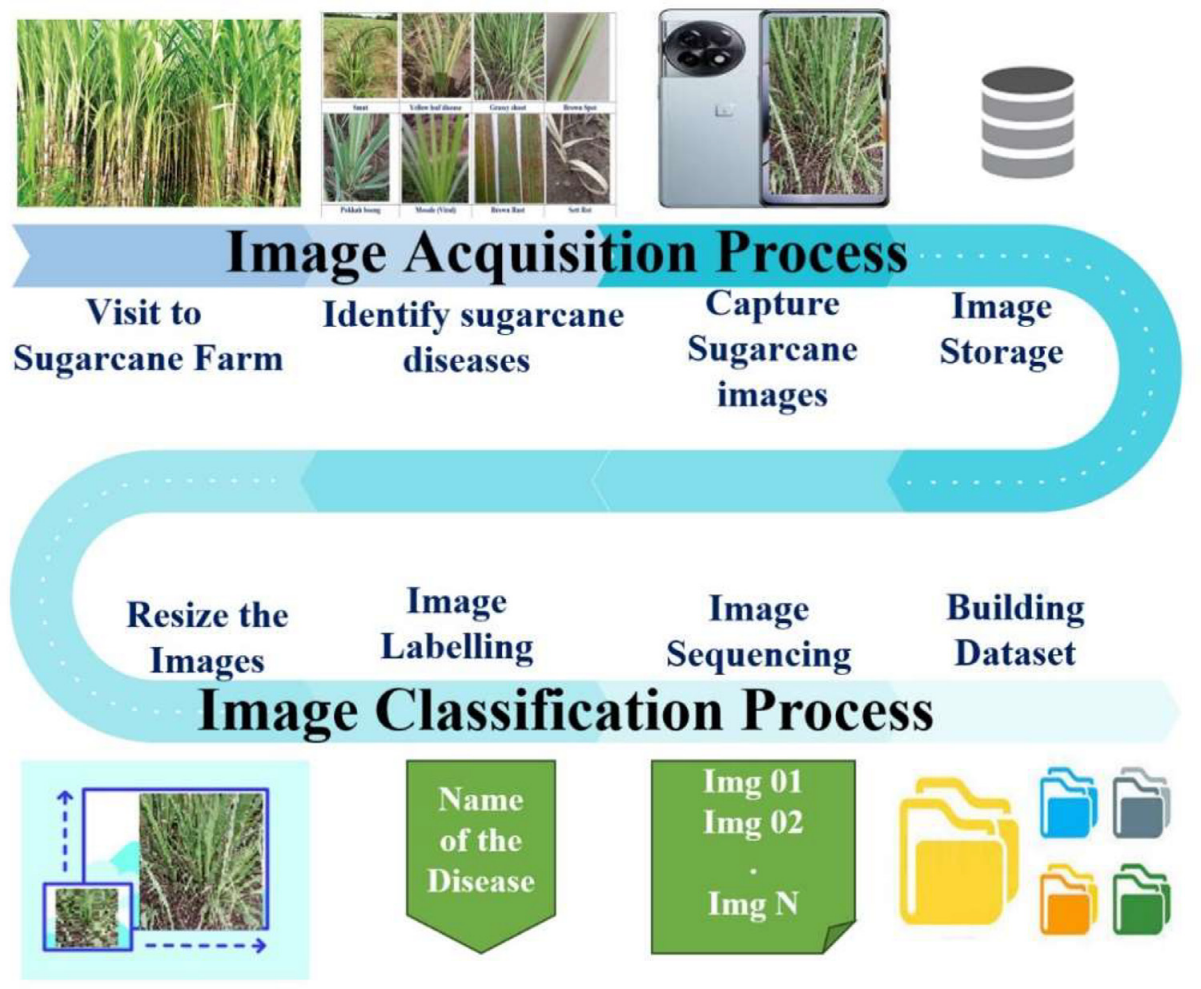
Architectural presentation of the image acquisition and image pre-processing.

The sugarcane leaf disease dataset was compiled through fieldwork conducted at a farm located in the village of Kendur, Taluka- Shirur, District -Pune, Maharashtra, India (geographical coordinates: 18°47′06.4″N 74°01′19.5″E). The data collection process involved capturing images under diverse scenarios, encompassing leaves within their natural habitat as well as leaves that had been detached or severed from the plant, all from a distance of 30–50 cm. This deliberate approach aimed to provide a comprehensive and varied representation of sugarcane leaf diseases under different environmental conditions. To ensure accurate disease identification, the collected images were forwarded to the Botany Department of Rashtrapita Mahatma Gandhi Arts and Science College in Nagbhid, Chandrapur, India. The department's expertise was leveraged to confirm the disease categories present in the images. Subsequently, the captured images underwent a pre-processing phase, which involved resizing and renaming, facilitated by the utilization of IrfanView software [4]. The resized and renamed images were systematically organized into folders corresponding to their respective disease categories. This curation process enhances the dataset's suitability for scientific analysis and research on sugarcane leaf diseases (Fig. 2).

Table 3 presents the distribution of images by various categories of sugarcane leaf diseases and healthy leaves in the dataset. The dataset consists of a total of 6748 images, with each category containing a different number of images. The categories include Yellow leaf disease (YLD), Smut, Pokkah boeng, Mosaic (Viral Disease), Grassy Shoot, Brown Spot, Brown Rust, Banded Chlorosis, Sett rot, Dried Leaf, and Healthy Leaves. The number of images for each category ranges from 246 to 1722. The original format of the images is now accessible to the public through Mendeley [5].

**Table 3:** Total number of images per category in the sugarcane leaf dataset.

Categories		Total Number of Images
Sugarcane Leaf Disease Name	Yellow leaf disease (YLD)	1194
	Smut	316
	Pokkah boeng	297
	Mosaic (Viral Disease)	663
	Grassy Shoot	346
	Brown Spot	1722
	Brown Rust	314
	Banded Chlorosis	471
	Sett rot	652
Dried Leaves		343
Healthy Leaves		430
**Total Number of Images in the Dataset**		**6748**

## Ethics Statement

Our study does not involve studies with animals or humans. Therefore, we confirm that our research strictly adheres to the guidelines for authors provided by Data in terms of ethical considerations.

## CRediT authorship contribution statement

**Sandip Thite:** Methodology, Data curation, Writing – original draft. **Yogesh Suryawanshi:** Conceptualization, Writing – review & editing. **Kailas Patil:** Conceptualization, Supervision, Writing – review & editing. **Prawit Chumchu:** Writing – review & editing.

## Data Availability

Sugarcane Leaf Image Dataset (Original data) (Mendeley Data).

## References

[1] Suryawanshi Y., Patil K., Chumchu P. (2022). VegNet: dataset of vegetable quality images for machine learning applications. Data Br..

[2] Kaur S., Pandey S., Goel S. (2019). Plants disease identification and classification through leaf images: a survey. Arch. Comput. Methods Eng..

[3] Hemalatha N.K., Brunda R.N., Prakruthi G.S., Prabhu B.B., Shukla A., Narasipura O.S.J. (2022). Deep Learning for Sustainable Agriculture.

[4] Suryawanshi Y., Gunjal N., Kanorewala B., Patil K. (2023). Yoga dataset: a resource for computer vision-based analysis of Yoga asanas. Data Br..

[5] PATIL Kailas, Suryawanshi Yogesh, chumchu prawit, Thite Sandip (2023). Sugarcane leaf dataset. Mendeley Data.

